# Lasting benefits of embryonic eavesdropping on parent-parent communication

**DOI:** 10.1126/sciadv.adn8542

**Published:** 2024-08-30

**Authors:** Francisco Ruiz-Raya, Alberto Velando

**Affiliations:** Centro de Investigación Mariña, Universidade de Vigo, Grupo de Ecoloxía Animal, Vigo 36310, Spain.

## Abstract

Developing embryos have traditionally been viewed as passive agents in the evolution of family conflicts, with maternal substances within the uterus or eggs as main factors modulating later expression of offspring solicitation behaviors. Yet, parent-offspring conflict theory predicts that offspring might also rely on alternative cues to adjust demand in response to prenatal cues of parental capacity for resource provisioning. Here, we show how embryonic experience with vocalizations carried out by parents during nest-relief displays at incubation adaptively shapes avian offspring development, providing lasting benefits to offspring. Genetic siblings prenatally exposed to different levels of parent-parent communication showed differences in epigenetic patterns, adrenocortical responsiveness, development, and food solicitation behavior. The correspondence between prenatal acoustic experience and parental context positively influenced the nutritional status and growth rate of offspring reared by communicative parents. Offspring can thus retain strong control over their own development by gathering prenatal acoustic information about parental generosity.

## Teaser

Prenatal experience with parent-parent communication shapes embryo development and matches offspring demand to parental care.

## INTRODUCTION

Family is a primary arena for cooperation and conflict (*1*). In species with biparental care, parents come into conflict as each will benefit better if the other bears a greater share of the costs (*2*). Nevertheless, breeding pairs often cooperate to raise their offspring, gaining additional resources by avoiding escalation of conflict (*3*). Parents are likewise expected to balance fitness gains from current and future reproductive attempts (*4*), which leads to differences in optimal strategies for parents and offspring over resource allocation [parent-offspring conflict; (*4*, *5*)]. This conflict is responsible for selective forces that generate an alignment between parental provisioning and offspring demand during early postnatal life (*6*–*8*), which may be the result of selection on parents or offspring depending on who controls access to information about future provisioning (*9*). Most research on this topic has focused primarily on maternal hormones acting in the prenatal environment (womb or eggs) as drivers of parent-offspring coadaptation (*9*), yet the active role of developing embryos in relying on external cues for information about parental capacity has been overlooked. Maternal hormone allocation and offspring outcomes may depend not only on the maternal ability to manipulate information sent to offspring but also on the offspring’s ability to rely on alternative cues on parental capacity (*10*, *11*). Here, we show how avian embryos collect external information about parental capacity for resource provisioning by eavesdropping on the vocal communication between their parents during nest-relief displays, which may allow offspring to adjust their development to the expected parental context.

Sound represents an accessible external cue to embryos of many species, making it a potential source of early information (*12*). Developing embryos have the potential to perceive, respond to, and learn acoustic signals from both their parents and the surrounding environment. In humans, the auditory experiences during the fetal period have a notable influence on infants’ neural processing and learning abilities (*13*), and prenatal exposure to certain types of speech, such as the maternal voice, can affect fetal development and postnatal behavior (*14*, *15*). Songbird embryos have been found to learn and recognize familiar sounds through habituation (*16*, *17*), and the effects of early auditory experiences may persist and shape offspring development and fitness (*18*–*20*). In zebra finches (*Taeniopygia guttata*), parental calls to eggs shape song learning and courtship behavior of juvenile males (*21*), as well as the thermal preferences of breeding adults (*22*). Sounds from the environment outside the eggs can affect avian embryo development through epigenetic and endocrine changes that occur during the late prenatal period (*23*–*25*), consistent with the timing of avian sensory development (*26*). Early acoustic experience affects genome-wide methylation in the auditory forebrain of late-stage songbird embryos (*24*). Similarly, prenatal acoustic stimuli alter whole-genome DNA methylation in seabird embryos (*23*), including methylation and expression patterns of specific target genes associated with chicks’ physiology and behavior [glucocorticoid receptor *Nr3c1* gene (*25*)].

Parental sounds are potentially informative for developing embryos and may act as anticipatory cues, indicating environmental features that will become relevant in subsequent life stages (*12*). For example, the characteristic calls produced by zebra finch parents while incubating eggs at high temperatures adaptively affect subsequent nestling growth and begging behavior, preparing offspring for high ambient temperatures (*22*). Embryos’ access to external acoustic information from parents may play a key role in parent-offspring conflict over parental care. Here, we hypothesize that vocal communication between parents during nest-relief displays might be used by avian embryos as an anticipatory cue about the parents’ capacity for resource provisioning. In socially monogamous species, displays between the sexes often continue after pair formation and extend through incubation or pregnancy (*27*). Such mutual displays include elaborated vocalizations that stimulate breeding partners to increase their investment in current reproduction (*28*, *29*). In some species, mutual vocalizations allow breeding partners to exchange information for effective coordination of parental activities, such as incubation or foraging behaviors (*30*–*32*). Parent-parent vocal communication during mutual displays between breeding partners might act as an acoustic cue on which embryos eavesdrop, informing about parents’ capacity and predisposition for parental care. Embryonic experience with vocal communication between parents could adaptively shape offspring development, leading to an alignment between offspring solicitation behavior and the parents’ capacity for care during early life.

In this study, we explored the effects of parent-parent vocal communication during nest-relief displays on offspring developmental trajectories, behavior, and postnatal performance in yellow-legged gull (*Larus michahellis*) families on the Sálvora Island, Spain. Large gulls (*Larus* sp.) are a model system for decades of research on maternal effects (*33*), signaling (*34*), and parental care (*35*). Gulls are ideal for our objective, as the elaborate mutual display rituals performed by sexual partners during courtship and egg incubation, as described by Tinbergen (*36*) more than half a century ago, offer a stark example of the precise and directed coordination of parental tasks. Gulls’ nest-relief rituals during incubation include vocal communication between pair members to which developing embryos in the nest are directly exposed. In *Larus* gulls, nest-relief displays during incubation may serve as a means of communication about foraging opportunities and contribute to the equal division of parental care (*37*), likely reflecting the compatibility of parental behavioral strategies. Communicative gull pairs coordinate incubation turn-taking and foraging patterns better, providing more care to eggs and chicks (*31*, *37*). Gull embryos have been found to be sensitive to acoustic stimulation during late embryonic development (*23*, *25*), yet whether parent-parent communication has the potential to match offspring demand to parental care remains unknown.

**Fig. 1.**
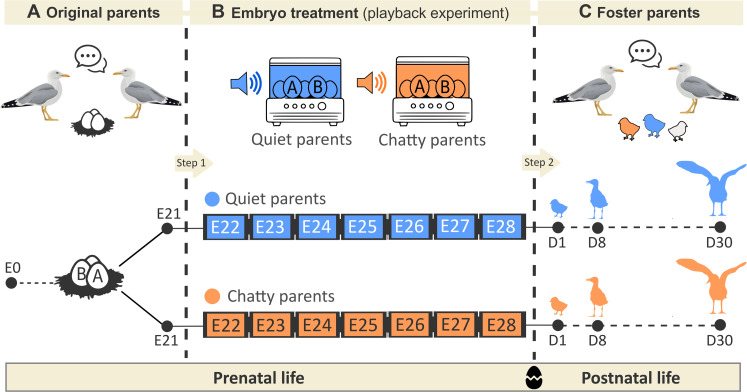
Diagram of the experimental setup. (**A**) Eggs were incubated in natural nests (i.e., by their original parents) until embryonic day 21 (E21). (**B**) From the embryonic day 22 (E22) to hatching, A (first-laid) and B (second-laid) eggs were artificially incubated at standard temperature (37.5°C). Siblings (A and B eggs from the same nest) were alternately assigned to different embryo treatments, so that they were periodically (three times a day) exposed to 2-min acoustic playbacks of either high (chatty parents) or low (quiet parents) levels of parent-parent vocal communication. The duration, intensity, and frequency of playback stimuli were within the natural range of variation in our study colony (see Materials and Methods). (**C**) Nestlings hatched in the incubators were individually marked and immediately returned to natural foster nests with a similar laying date (step 2). Chicks were cross-fostered between our 44 original families, in which we recorded their levels of parent-parent communication. Original siblings were reared (together) by the same foster family (plus an unmanipulated C chick—white chick in the figure—simulating a natural three-egg clutch scenario) so that each foster family reared two chicks that differed in their embryo treatment. Chicks were blood sampled at day 1 posthatching, and their tarsus length and body mass measured at days 1 (D1), 8 (D8), and 30 (D30) of age. Begging tests were individually conducted on each chick at D1 to assess solicitation behavior (see Materials and Methods).

We began by characterizing the levels of parent-parent vocal communication during nest-relief behavior at incubation in 44 gull families with three-egg clutches. In parallel, we manipulated the exposure of sibling embryos to parent-parent vocal communication using playback treatments (communicative, “chatty” parents versus noncommunicative, “quiet” parents) in artificial incubators (see Fig. 1 for a schematic representation of the experimental setup). We explored the epigenetic and physiological mechanisms that may allow embryonic experiences with parental sounds to persist and shape future phenotypic outcomes (*38*). Prenatal exposure to conspecific acoustic stimuli is known to result in lower genome-wide methylation levels in avian embryos, which may translate into downstream effects on transcriptional activation and gene expression patterns (*24*). Similarly, if parent-parent vocal communication during displays is informative on the levels of parental attentiveness (*29*, *31*, *37*), then it could be expected changes in the adrenocortical response to stress (*39*). We also tested differences in begging behavior in newly hatched chicks exposed to different embryo treatments (chatty versus quiet parents). According to our hypothesis, chicks prenatally exposed to cues of chatty parents are predicted to show increased begging behavior, which would align solicitation strategies and parental capacity for resource provisioning.

We then cross-fostered newly hatched chicks between the original gull families, which largely varied in the level of parental vocal communication (match-mismatch manipulation). This allowed us to assess the long-term performance of siblings raised in the same family context but differing in their prenatal experience with parent-parent vocal communication. We examined individual chick performance (growth rate and nutritional condition) during the first week of life, a critical period of rapid growth during which offspring are completely dependent on their parents for both food and protection (*40*). In addition, we tested whether the interactive effects of prenatal acoustic cues and foster parents’ context on offspring performance lasted until the fledging stage. We predicted that the correspondence between prenatally (acoustically) induced offspring phenotypes and the postnatal parental context will bring adaptive benefits to offspring, as predicted by a coadaptation theory (*6*, *8*, *9*).

## RESULTS

### Associations between parent-parent vocal communication and parental care in gulls

Video monitoring of gull pairs confirmed that families vary greatly in their levels of parent-parent vocal communication during incubation, ranging from pairs in which nest reliefs occurred without any vocalization to pairs that engaged in repeated and ritualized social interactions accompanied by long vocalizations (fig. S1A; see movies S1 and S2). There is strong evidence for the key role of vocal communication between parents for parental duties. In many bird species, including gulls (Laridae), mutual vocal communication between parents during incubation is a reliable proxy of breeding pair coordination or capacity for parental care, informing on the relative willingness of pair members to perform parental tasks (*32*, *37*). In our gull pairs, we found a negative relationship between the duration of parent-parent vocal communication and the proportion of time that the nest was left unattended (χ^2^ = 5.06, df = 1, *P* = 0.024). Previous research has found that communicative parents attend more both eggs and nestlings than do noncommunicative parents, mainly due to enhanced distribution of parental duties (*30*–*32*).

### Prenatal experience with parent-parent communication shapes offspring development and solicitation

We experimentally manipulated the exposure of developing embryos to parental vocalizations. To do that, we collected the first two eggs [i.e., A and B eggs, with similar maternal background (*41*)] from our 44 three-egg clutches (approximately 8 days before hatching) and maintained them in artificial incubators during the late incubation period (Fig. 1). Recent evidence shows that gull embryos are sensitive to acoustic stimulation in this late developmental period, with important consequences on development (*23*, *25*). We assigned sibling embryos to different playback treatments, which mimicked two different scenarios (duration) of parent-parent vocal communication (within a natural range). Every day until hatching (from incubation day 21 to day 28; see Fig. 1), we periodically exposed one of the two sibling embryos to acoustic cues of communicative parents (“chatty parents”; *n* = 44) and the other to acoustic cues of noncommunicative parents (“quiet parents”; *n* = 44). Egg volume, laying date, and embryo sex did not differ between the experimental treatments (*P* > 0.62 in all cases).

**Fig. 2.**
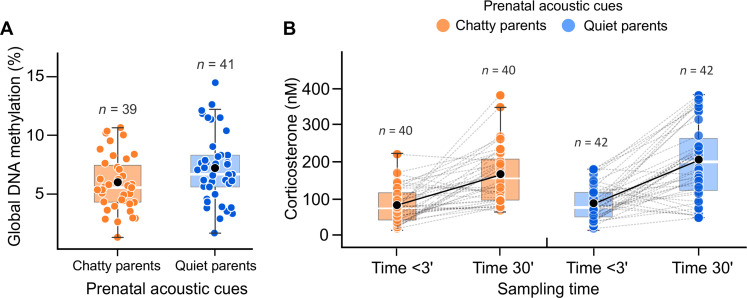
Prenatal exposure to different levels of parent-parent communication has an impact on offspring epigenetic patterns and adrenocortical responses. (**A**) Hatchlings prenatally exposed to playback of quiet parents showed increased global DNA methylation levels compared to hatchlings exposed to playback of chatty parents. (**B**) Hatchlings prenatally exposed to chatty parents showed a less pronounced increase in plasma CORT levels in response to a standardized stressor (capture followed by 30-min restraint) compared to hatchlings exposed to quiet parents. Points show raw data; inset boxplots depict medians and center quartiles; black points indicate mean values.

We found that the level of parental communication had a strong programming effect on embryonic development. Embryos exposed to chatty-parent playbacks showed a longer embryo developmental period than those exposed to quiet-parent playbacks [β = −0.43, 95% confidence interval (CI): −0.80, −0.08, *P* = 0.015; table S1 and fig. S2]. Prenatal exposure to different parental communication also had effects on major epigenetic and endocrine mechanisms controlling vertebrate development (*42*). Specifically, 1-day-old hatchlings exposed to chatty-parent playbacks during the late embryonic period showed reduced DNA methylation marks in their genome compared to those exposed to quiet parents (β = 0.47, 95% CI: 0.03, 0.90, *P* = 0.032; table S1 and Fig. 2A). Our embryo treatment also affected the hatchling adrenocortical response to a standardized stress protocol (capture followed by 30-min restraint; see Materials and Methods) as chicks prenatally exposed to chatty parents showed reduced corticosterone (CORT) reactivity compared to those of quiet parents (embryo treatment × sampling time: β = 0.46, 95% CI: 0.02, 0.89, *P* = 0.039; table S1 and Fig. 2B).

**Fig. 3.**
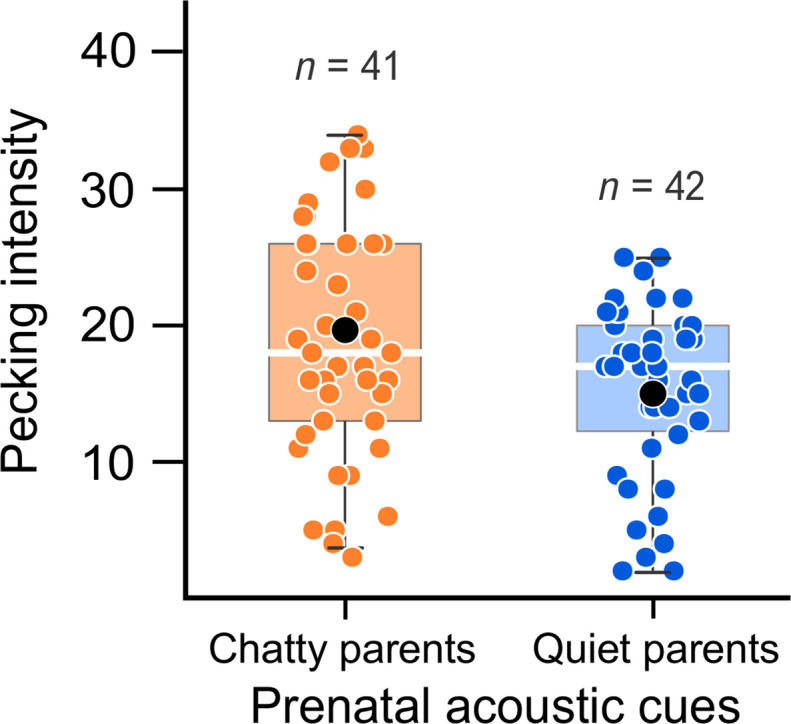
Effects of prenatal acoustic treatment on offspring begging behavior. Hatchlings prenatally exposed to playbacks of chatty parents showed higher begging intensity (increased pecking behavior) compared to those that received playbacks of quiet parents. Points show raw data; inset boxplots depict medians and center quartiles; black points indicate mean values.

We also assessed whether the exposure of gull embryos to parent-parent vocal communication shapes fitness-relevant traits, such as food solicitation strategies during the nestling period. Using standardized individual behavioral tests, we evaluated the intensity of newly hatched chicks’ begging behavior (pecking behavior, a crucial component of gull chick solicitation behavior), following a previously established protocol (*43*, *44*). Chicks prenatally exposed to chatty parents’ playback showed higher pecking rates than did those of quiet parents (β = −0.54, 95% CI: −1.03, −0.04, *P* = 0.022; table S1 and Fig. 3). Brood hierarchy (first or second hatched) did not influence begging behavior (*P* = 0.575; table S1). The interaction between the genetic parents’ vocal communication and embryo treatment was not significant in any of our models (all *P* > 0.542).

### Offspring prenatally exposed to parent-parent communication perform better when reared by communicative parents

To test the adaptive value of experimentally induced offspring phenotype, we conducted a match-mismatch manipulation by cross-fostering the experimental hatchlings between original families. Briefly, siblings that received different treatments during the late embryonic period were individually marked and placed together, immediately after hatching, in a natural foster nest with a similar laying date. Each foster gull pair reared two siblings from the same original parents but received different embryo treatments (within-nest manipulation; Fig. 1C). Exchanging young between parents allowed us to disrupt the correlation between offspring solicitation and parent provisioning, as well as prenatal hormone signaling (*9*). We expected that the correspondence between offspring phenotype and postnatal parental context (capacity for resource provisioning) will bring adaptive benefits to the offspring (*6*, *8*, *9*).

**Fig. 4.**
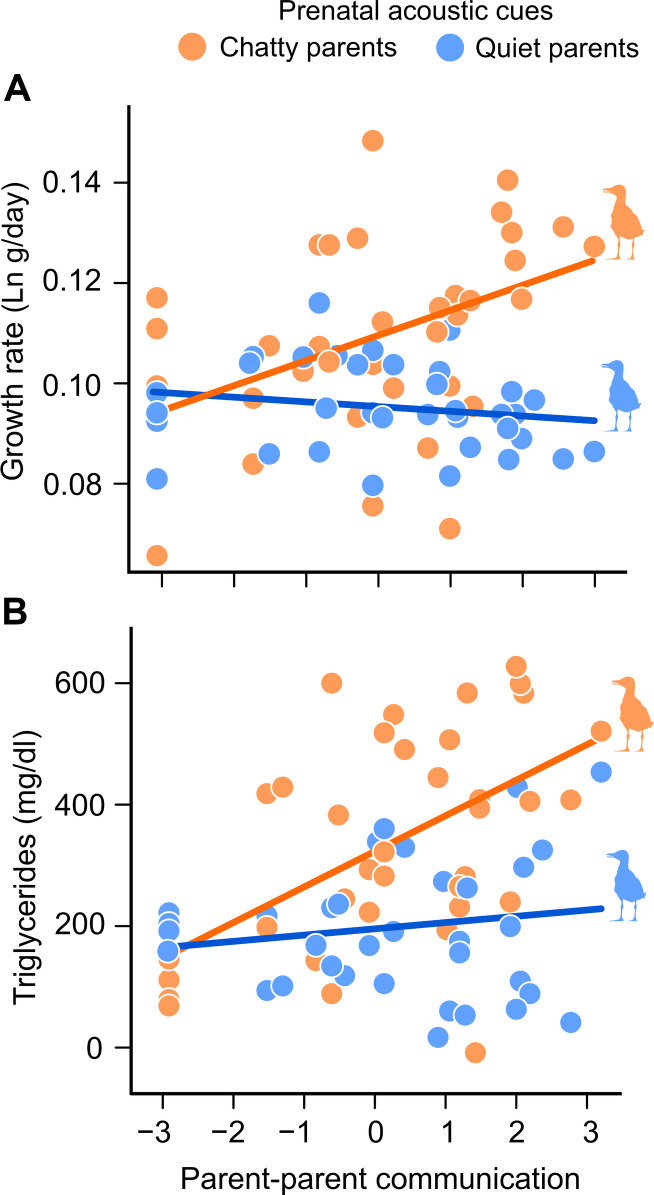
Prenatally cued offspring performed better during early postnatal development (8 days after hatching) when reared by chatty parents. (**A**) Offspring prenatally exposed to high levels of parental vocal communication (chatty parents) exhibited a higher growth rate than their siblings when reared by chatty parents. (**B**) Offspring that received prenatal acoustic cues reporting high levels of parental communication (chatty parents) showed improved nutritional status in terms of plasma triglyceride levels when reared by chatty parents. Mean-centered values for parent-parent communication are shown.

Chick growth rate during the first week was positively related to the level of (foster) parent-parent vocal communication, but only in those prenatally exposed to chatty parents’ playback (embryo treatment × foster parent communication: β = −0.41, 95% CI: −0.75, −0.06, *P* = 0.020; table S2 and Fig. 4A). This result indicates that chicks prenatally programmed by acoustic cues of communicative parents obtained more food resources (or better resources in quality) from chatty parents in comparison to their siblings programmed by cues of quiet parents but reared by the same foster parents. Only those chicks that received prenatal cues from chatty parents showed improved nutritional status in terms of plasma triglyceride concentration when reared by chatty pairs (embryo treatment × foster parent communication: β = −0.45, 95% CI: −0.81, −0.09, *P* = 0.019; table S2 and Fig. 4B). In gull chicks, high plasma triglyceride levels typically mirror lipid-rich prey provided by parents (*45*), and lipid-poor diets often cause nutritional stress, reduced growth, and increased mortality in seabirds (*46*, *47*). In our study, growth rates were indeed positively correlated to lipid profiles in 8-day-old chicks (*r* = 0.42, *P* < 0.0001, *N* = 69; fig. S3). Chick’s triglyceride level was positively related to the level of parent-parent vocal communication of original (genetic) parents (β = 0.24, 95% CI: 0.03, 0.44, *P* = 0.012; table S2 and fig. S4). It is possible that females in highly communicative (chatty) pairs had lipid-rich nutrition, thereby producing eggs with increased lipid levels in yolk, which can directly affect the nutrient reserves available to the developing chicks (*48*). Begging intensity at hatching was positively associated with chick growth during the first 8 days after hatching (β = 0.33, 95% CI: 0.13, 0.52, *P* = 0.001; table S2 and fig. S5). The interaction between the genetic parents’ vocal communication and embryo treatment, or foster parent communication, was not significant in the growth rate and plasma triglyceride models (all *P* > 0.456).

### Long-lasting benefits of embryo exposure to parent-parent vocal communication

**Fig. 5.**
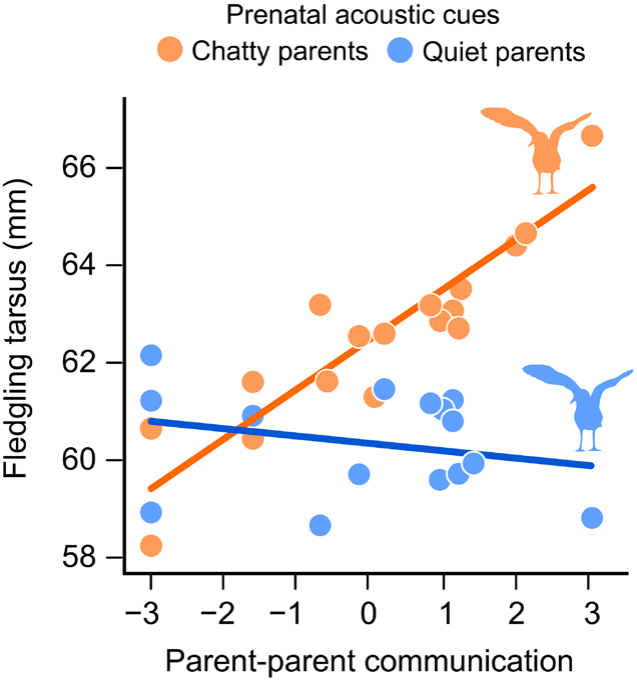
Prenatally cued offspring showing lasting benefits of embryonic eavesdropping (30 days after hatching) when reared by chatty parents. Body size (fledgling tarsus length) at day 30 posthatching was positively related to parental communication levels, but only for those chicks that were prenatally exposed to vocalizations of chatty parents. Mean-centered values for parent-parent communication are shown.

At 30 days posthatching, both body size (tarsal length; embryo treatment × foster parent communication: β = −0.49, 95% CI: −0.77, −0.20, *P* = 0.002; table S3 and Fig. 5) and mass (embryo treatment × foster parent communication: β = −0.25, 95% CI: −0.47, −0.04, *P* = 0.009; table S3 and fig. S6) were positively related with the level of foster parent communication but only in those fledglings that received prenatal acoustic signals of chatty parents. Since skeletal size at this age is maintained until adulthood, the beneficial effect of matching prenatal cues and parental quality is expected to extend to adulthood (*49*). The interaction between the genetic parents’ vocal communication and embryo treatment, or foster parent communication, was not significant in the body size and body mass models (all *P* > 0.245).

These results highlight that prenatal experiences with parents during the embryonic development of acoustic sensitivity trigger changes that persist throughout the prenatal and postnatal development and adaptively shape the phenotypic outcomes of offspring. In our study, siblings with similar genetic and maternal backgrounds were raised by the same foster parents, so they only differed in the acoustic signals that they received during a brief window of embryonic sensitivity. It is possible that acoustically induced changes in DNA methylation and the hypothalamic-pituitary-adrenal axis during this embryonic period promote stable behavioral phenotypes in gulls (*23*, *25*). Prenatal experience with parent-parent vocal communication during this brief window affected the developmental trajectories, inducing proactive chicks’ high begging rates, which were favored by chatty pairs until the fledgling stage. Future studies should evaluate whether the benefits associated with these adjustments persist and play an important role in individual success during adult life.

## DISCUSSION

We found that avian embryo exposure to parent-parent vocal communication affects genome-wide methylation and adrenocortical response to stress and shapes offspring development and solicitation strategies. Our cross-fostering manipulation revealed the role of parent-parent vocal communication in promoting offspring adaptation to parental capacity, as evidenced by the different performances of genetic siblings (i.e., with similar maternal backgrounds) exposed to different prenatal acoustic cues but then reared in an identical family environment. These results suggest that, at least in gull chicks, the prenatal matching of offspring solicitation to parental supply (parent-offspring coadaptation) may be mediated by prenatal experience with parent-parent vocal communication, which may inform offspring about parents’ capacity to provide resources beyond hormone-mediated maternal effects (*9*). These findings may help to elucidate whether parents or embryos control developmental outcomes and therefore the evolutionary consequences of maternal effects.

The early-life environment may contribute to permanent and long-lasting effects on individual phenotypes in animals, with prenatal life being a critical window for epigenetic effects allowing embryonic experiences to persist after birth or hatching (*38*, *50*). In our study, global DNA methylation and stress response measured soon after hatching differed between the treatments, indicating prenatal adjustments in these control mechanisms. In birds, much of the genome-wide DNA methylation is established during late embryonic development (*50*), and individual adrenocortical stress responses can be particularly sensitive to environmental conditions during this critical developmental window. It has recently been shown that gull embryo exposure to external environmental cues of postnatal stressors increases global DNA methylation (*23*) and promotes CORT reactivity (*51*), with consequences on behavioral phenotypes of newly hatched chicks (*23*, *51*). Accordingly, we found increased DNA methylation and CORT reactivity in hatchlings prenatally exposed to cues of noncommunicative (quiet) parents. While the specific consequences of these changes still need to be established, our results suggest changes in developmental programs presumably governing the development of postnatal phenotypes (*42*).

Prenatal acoustic experience with parent-parent vocal communication also affected fitness-relevant traits such as offspring solicitation strategies. Specifically, chicks prenatally exposed to acoustic cues of communicative parents showed higher begging intensity than did those of quiet parents. This suggests that the prenatal experience with parent-parent communication during nest-relief displays does contribute to natural variation in gull begging behavior, with prenatal perception of high parental care (chatty parents) leading to increased food solicitation. Since full siblings from the same clutch received different prenatal acoustic treatments, our findings indicate that embryonic eavesdropping on parental communication does mediate plasticity in the expression of offspring solicitation behaviors beyond genetic (*52*) and hormone-mediated maternal effects (*7*, *9*). Thus, prenatal exposure to parent-parent vocal communication could be expected to have anticipatory programming effects (*49*), with the potential to facilitate correspondence between offspring solicitation strategies and parental food provisioning. As shown by experimental work and theoretical models, displays between breeding pairs may stimulate the partner to increase investment in offspring, allowing sexual conflict to evolve into a stable cooperative system and strengthening the pair bond (*28*, *29*). Parent-parent communication can equally allow parents to exchange information for effective coordination of incubation and offspring provisioning (*30*, *31*). Irrespective of the mechanism, our and previous results suggest that communicative (chatty) gull parents have the potential to provide more care to offspring.

Gull chicks’ growth rate and nutritional status were positively correlated with levels of intrapair communication shown by foster parents, albeit exclusively in those chicks that were prenatally exposed to acoustic cues of chatty parents. Intrapair communication during nest-relief ceremonies may play a crucial role in the coordination of parental incubation, foraging, and provisioning strategies (*53*), especially in seabirds undertaking long foraging trips (*54*, *55*). Effective foraging strategies of breeding pairs for high-quality preys (lipid-rich fish) are expected to increase parental resources for their offspring (*56*, *57*). In our study, the benefits of prenatal eavesdropping on parent communication were particularly evident when offspring were reared by chatty parents, probably because they were able to provide high-quality nutritional food or respond to increased solicitation behavior. Chicks’ pecking behavior does elicit parental provisioning in gulls (*34*, *43*), and food solicitation intensity was positively associated with chick growth. Experimental chicks that were exposed to chatty parents’ playback begged more intensively, and this probably allowed them to gain extra resources from highly communicative parents. Since both siblings were raised by the same foster parents, our results suggest that communicative parents allocated more or higher-quality food to prenatally cued offspring (with higher solicitation rates). Future research should aim to gain a deeper understanding of the physiological and molecular mechanisms that underlie offspring developmental changes in response to parent-parent communication and to determine how the resulting offspring phenotype influences parental provisioning strategies.

Access to information has the potential to influence the outcome of family conflicts (*1*). Maternal substances have been proposed to play a key role in parent-offspring conflict by promoting correlation (coadaptation) of offspring solicitation strategies and parental supply. Parent-offspring coadaptation may result from selection on either the parent or the offspring, depending on who controls access to information about future provisioning (*9*). In our study, vocal communication of original (genetic) parents did not modulate later expression of offspring solicitation behaviors, and the match between the communication of original and foster parents did not affect offspring performance. The consequences of experimentally disrupting the correlation between parental supply and offspring demand by exchanging young between parents suggest a minor role, if any, for maternal effects in offspring adaptation to parental provisioning in gulls, where provisioning rules are mainly controlled by parents (see movie S3) (*23*). Under these circumstances, it can be predicted that external information about parental quality, such as that provided by parent-parent vocal communication, would favor the match between offspring solicitation and parental supply (*9*), as occurs in our study. Under natural conditions, clutch mates would receive identical acoustic information from their parents, so a possibility is that this information influences cooperation and coadaptation in a whole family context (i.e., the correspondence of brood demands and parental provisioning). While future studies are needed to confirm this prediction, it is plausible that the higher reproductive success shown by communicative gull pairs found in previous studies (*31*) is linked to prenatally induced proactivity of offspring in concert with efficient parenting within these families.

Our results indicate that parent-parent vocal communication provides unborn offspring with important information about potential parental care beyond direct signals conveyed through endocrine and nutritional maternal effects. The prominence of parental sounds during embryonic development makes parent-parent vocal communication a particularly informative cue for offspring regarding parental generosity, which has important implications for the evolutionary conflict between parents and offspring over resource allocation (*58*). Because animals are expected to integrate different sources of information to produce an adaptive response to variable environments (*59*), developmental trajectories should rely on accurate estimation of the parental context in which offspring will develop. Prenatal acoustic communication may allow embryos to update information about the postnatal environment and adjust their development and behavior accordingly, especially when external cues do not match expectations based on other sources of information (e.g., components of the maternal egg). By eavesdropping on parental vocal communication during embryonic life, offspring would benefit by bypassing parental influence and optimizing their phenotypic trajectory (*10*). This challenges the traditional view of developing embryos as a passive agent in parent-offspring conflict and highlights the importance of recognizing the often-overlooked active role of the embryo in family evolutionary interactions (*58*).

## MATERIALS AND METHODS

### Study system

We conducted our study in a yellow-legged gull (*L. michahellis*) colony on Sálvora Island (Spain) during May to June 2021. At the beginning of the breeding season, we surveyed the study area daily and marked gull nests with numbered sticks. We checked the gull nests daily (09:00 to 12:00 hours) until clutch completion, and newly laid eggs were individually marked using a nontoxic marker to register laying order. Yellow-legged gulls (gulls hereafter) typically lay eggs at 1- to 3-day intervals until the clutch is completed (modal clutch size = three eggs). Egg incubation usually begins after the second egg is laid, and eggs hatch asynchronously after, on average, 28 days of incubation (*23*).

### Natural levels of parent-parent vocal communication in gull families

In many birds, including gulls, vocal communication between parents during incubation is considered a reliable indicator of parental care, as communicative parents incubate longer, reduce nest relay costs, and take better care of eggs and chicks due to better sharing of parental duties (*30*, *31*, *37*). We assessed the natural levels of parent-parent vocal communication during late incubation [when avian embryos are expected to be sensitive to acoustic stimulation (*26*)] in 44 three-egg gull families. Video cameras (Victure HC200) were placed at a 4- to 5-m distance from the focal nests (on partially camouflaged wooden supports) on day 24 of incubation. A wooden support, in addition to a wooden piece of similar dimensions to the video camera, was placed at a 4- to 5-m distance from the focal nest 24 hours beforehand to habituate gulls to the presence of an unfamiliar object near the nest. On the day of filming (approximately 8:15 hours), we replaced the wooden piece by a camera previously programmed to be triggered by movement (typically during the arrival of individuals at the nest during incubation reliefs) and to film the parents’ behavior for 60 s. Several consecutive recordings were done in those cases when the nest relief lasted more than 60 s. We removed cameras at approximately 20:30 hours, resulting in 12.17 ± 0.03 hours of continuous monitoring. During this time, both parents coincided at the nest on 2.4 ± 0.23 occasions (fig. S7A), of which 1.8 ± 0.14 eventually resulted in nest relief (fig. S7B). Behavioral analyses of recordings were performed using Solomon Coder software. We quantified all parental vocalizations when both parents were present at the nest by scoring calls that have been associated with pair formation and cooperation in gulls, typically long calls, mew calls, and chocking [see (*60*) for a detailed description of these displays]. Following a previously established methodology for the analysis of parent-parent communication in gulls (*31*), we measured the duration of parental vocalizations (in seconds) from the start of each call to its end, and values were summed for each pair during the filmed period (mean ± SE = 71.8 ± 10.6 s; fig. S1A; see the Supplementary Materials for videos of parent-parent vocal communication during nest relief). Incubation behavior tends to be consistent throughout the incubation period in gulls (*31*, *61*), as does parent-parent vocal communication during incubation displays (*31*). Still, to assess the repeatability of parent-parent vocal communication in our study population, we video-monitored a subset of our nests (*N* = 35) again on incubation day 26 (i.e., 2 days after our first measure). Bootstrapping analyses (see the “Statistical analyses” section) confirmed that levels of parent-parent vocal communication [the duration of parent-parent communication (*31*)] were highly repeatable within experimental pairs throughout late incubation (*R* = 0.594 ± 0.12 [0.314, 0.777], *P* = 0.0002; fig. S1B).

In addition to monitoring parent-parent vocal communication in gull families, we placed light data loggers (AX3’s Axivity Data loggers; Axivity, United Kingdom) in 25 of our experimental nests (simultaneously with video monitoring) to assess changes in the intensity of sunlight received by the clutch during incubation, a reliable proxy of incubation constancy (*51*). Egg exposure to sunlight is known to vary between bird clutches depending on differences in parental incubatory constancy (*62*). Data logger devices (3 cm by 2 cm by 0.6 cm) were fixed to a support and nailed to the nest floor to avoid unintended movements as a result of parents’ behavior (fig. S7C). Light data (sunlight luminous intensity; lux) were recorded for twelve consecutive hours (one measure per second) and stored in the internal memory. We standardized light data according to specific light conditions in each nest (0 = complete darkness; 1 = maximum intensity). Then, we estimated the proportion of time that eggs remained over 50% of maximum light intensity: A conservative light threshold that unambiguously indicates the clutch is unattended (*51*). Data were processed using Open Movement GUI software v.1.0.0.43.

### Embryo treatment: Acoustic stimulation of developing embryos

Simultaneously with the analysis of natural levels of parent-parent vocal communication in gull families, we performed a playback experiment in which we manipulated (within a natural range) the exposure of developing embryos to parent-parent vocal communication (parent vocalizations) to assess its effects on the developmental trajectories and postnatal phenotypes of gull offspring. Avian embryos are particularly sensitive to acoustic stimulation several days before hatching (*26*) and, specifically, from embryonic days 21 to 22 of incubation in large *Larus* gulls (*23*). On day 21 of incubation, both A and B eggs [i.e., genetic siblings with similar maternal background (*41*); *N* = 88] from our 44 gull families were collected (20:00 to 21:00 hours) and immediately transported to our field station (<800 m distance) in an opaque thermal container. Natural eggs were replaced by fake gull eggs to maintain three-egg clutches in our experimental nests. Once in the field station, eggs were artificially incubated at a standard temperature (37.5°C; six incubators; fig. S7D).

Every day between days 22 and 28 of incubation (embryonic days 22 to 28), we collected the eggs from artificial incubators and placed them inside a sound-proof box (40 cm by 30 cm by 30 cm), which were exposed to playback stimuli of parent-parent vocal communication (playback trials) that were previously recorded in the breeding colony. To determine the most appropriate periodicity, duration, and sound intensity of playback trials, we previously conducted a short pilot study in an area near our experimental nests to assess the natural levels of parental communication in gull nests. Briefly, we filmed the behavior of nine gull pairs on incubation days 24 to 26 (8:00 to 20:00 hours) by using video cameras (Victure HC200; see the procedure above). On average, both parents coincided at the nest on 2.8 ± 0.4 occasions (range, 1 to 5), of which 2.1 ± 0.4 resulted in nest relief (range, 1 to 4). The mean duration of parent-parent communication for the recording period was 69.4 ± 23.0 s (range, 2 to 216 s). In addition, we recorded the sound intensity of parent vocalizations in the nest (in decibels) at ground level by using the mobile app “dB SoundMeter” (Pony Inc.; device partially camouflaged in the vegetation approximately 50 cm from the focal nest). The mean intensity of parental vocalizations at the ground level was 83.3 ± 1.0 dB (range, 77 to 88 dB).

The audio files used for acoustic stimulation during playback trials were broadcasted with a speaker (Carrefour bsp30) that was placed in the lid of the sound-proof box, positioned 30 cm from the eggs, and set to a standard sound intensity of approximately 82 dB (average, 2 min). Siblings (A and B eggs from the same nest) were alternately assigned to different embryo treatments, so that they were exposed (three times a day) to 2-min acoustic playbacks of either high (chatty parents, *N* = 44) or low (quiet parents, *N* = 44) levels of parent-parent communication. Eggs in the chatty parents group were exposed to playback stimuli three times a day between 09:00 and 20:00 on a random schedule to avoid habituation and using a different 2-min audio file each time from a subset of three files. The audio files used in these chatty parents playback trials included 70 s of parent-parent vocalizations (long calls, mew calls, and chocking) plus 50 s of background noise (that is, the sound produced by the speaker when it is switched on and background colony noise, typically birds in the distance, including sporadic songbird trills). In the quiet parents, gull eggs were subjected to the same experimental procedure of 2-min playback trials (three times a day), but they only received 10 s of parent-parent vocal communication plus 110 s of background noise in each playback trial. The duration (total per day, 210 and 30 s for the chatty parents and the quiet parents group, respectively), intensity (approximately 82 dB), and frequency (three times a day) of embryo exposure to cues of parent-parent vocal communication were within the natural range of variation in our study colony (see above). After each playback trial, the eggs were immediately returned to incubators. Egg volume, laying date, and embryo sex did not differ between the experimental treatments (*P* > 0.62 in all cases).

### Cross-fostering manipulation and postnatal offspring performance

Nestlings hatched in the incubators were individually marked with numbered leg flags and immediately returned to the colony to be reared in three-egg foster nests, mimicking natural conditions. Newly hatched chicks were cross-fostered between our 44 original families (of which we knew their levels of parent-parent communication; see above) to disrupt any potential covariation between parental and offspring phenotype. Chicks were cross-fostered between original families with a similar laying date (±1 day). Exchanging young between parents allowed us to disrupt any correlation between offspring solicitation and parent provisioning, as well as prenatal hormone signaling (*9*). Genetic siblings were reared together in the same family so that each foster pair reared two siblings that differed in their embryo treatment (chatty parents versus quiet parents). Five experimental eggs (5.7%; *N* = 88) did not hatch, but hatching probabilities did not differ between embryo treatments (χ2 = 0.004; df = 1; *P* = 0.95). At day 27 of incubation, nests were fenced with semitransparent plastic mesh (2-m diameter; 30-cm height) to avoid newly hatched gull chicks escaping from their territories (*44*). The fences were removed on day 8 posthatching, and all chicks were ringed with numbered plastic rings. The growth rate per day during the 8 days after hatching (the period of maximum growth) was calculated for all chicks as (lnW2 − lnW1)/T2 − T1, where W2 and W1 represent chick weight at day 8 and day 1, respectively, and T2 and T1 represent sampling days 8 and 1, respectively. At day 30 posthatching, when chicks were fully grown and near fledging, we came back to the colony and searched for them around their territories. We measured the chicks’ tarsus length (±0.1 mm) and body mass (±1 g) using a digital caliper and a Pesola spring balance, respectively, at days 1 (*N* = 83), 8 (*N* = 70), and 30 posthatching (*N* = 32). Differences in sample sizes reflect missing values due to death or loss of chicks; chick death/disappearance at day 30 posthatching did not differ between embryo treatments (χ2 = 0.290; df = 1; *P* = 0.59).

### Begging tests

Yellow-legged gull chicks show a complex begging behavior that has specific functions in both parent-offspring communication and sibling competition. When parents arrive at their territories from foraging, gull chicks typically peck at the red spot on the parent’s bill to stimulate food regurgitation (*63*). The number of pecks and parental provisioning (feeding rates) has been found to be highly correlated (*64*). Begging tests were individually conducted on each chick by following a well-established methodology (*43*), with minor modifications (*44*, *65*). One-day-old chicks were individually collected from their nests and transported in bird-holding bags to a hide placed outside the dense gull colony to avoid disturbance to the chick behavior by adult gulls’ alarm calls. Once there, we placed each focal chick on the ground and covered it with a cloth until it stayed calm and quiet. Then, the chick received a playback of three mew calls (previously recorded at the study colony) simulating a parental feeding event. Immediately after the mew calls, we removed the cloth and started presenting a dummy mimicking an adult gull’s head [see (*65*) for additional details on dummy presentation]. The presentation of dummies that simulate the head of a parent gull is known to elicit the begging behavior used by gull chicks to stimulate parents to regurgitate food (*43*, *65*). We performed the visual stimulation by nodding the dummy head close to the chick once every 2 s for 1 minute. In each begging test and blind to the treatment, we recorded the number of pecks delivered by the focal chick to the red spot during dummy presentation. Immediately after completing the begging test, we returned the chicks to their foster nests in the colony to be reared under natural conditions.

### Blood sampling and standardized stress protocol

We took 1-day-old chicks directly from the foster nest and collected a blood sample (∼90 μl) from the brachial vein immediately after capture using 80-μl heparinized microhematocrit tubes. All captures occurred between 08:00 and 11:00 hours to minimize variation in circulating plasma CORT associated with circadian rhythms. In all cases, initial blood samples were collected within 3 min (to reduce the possible effects of handling on CORT levels), so they were considered to reflect “baseline” CORT levels. Afterward, we followed a standardized capture-restrain technique simulating a stressful event to assess the CORT “stress response” (*66*, *67*). Briefly, after the first blood sample was collected, chicks were kept in an individual cloth bag and suspended off the ground outside the colony. We collected a second blood sample from the same individual (from the brachial vein of the opposite wing) 30 min after capture. Plasma CORT levels typically increase after individuals experience a stressor, reaching maximal concentrations within 30 to 60 min, so the second blood sample can be considered to reflect the CORT stress response [i.e., “stress-induced” CORT levels; (*67*)]. Blood samples were kept cold (2° to 8°C) and transported to our field station, which were centrifuged at 4000 rpm for 6 min (maximum of 3 hours after collection) to separate plasma from red blood cells. Plasma samples were immediately stored in liquid nitrogen. Once in the lab, all samples were stored at −80°C until molecular/hormonal assays.

### CORT levels

We quantified plasma CORT concentration using a commercially available enzyme-linked immunosorbent assay (ELISA Kit EIA-4164; DRG, Germany) and following the manufacturer’s instructions. Briefly, plasma samples (20 μl) were incubated with a CORT–horseradish peroxidase conjugate for 60 min in a flat-bottom microtiter plate. Then, the microtiter plate was washed three times and allowed to react with a substrate solution. The reaction led to a blue-green complex whose absorbance (450 nm; Synergy 2 Multi-Mode Microplate Reader, BioTek Instruments Inc.) was reversely proportional to the concentration of plasma CORT in the sample. Samples of the same individual were included on the same assay, with samples of different treatments randomized across plates. The CORT levels of all samples were above the detection limit (1.680 nM). All samples were analyzed in duplicate in five plates, with an average intra- and interplate coefficient of variation (CV) of 6.9 and 12.7%, respectively.

### Triglyceride levels

We measured triglyceride levels in all plasma samples on day 8 using commercially available kits (Biosystems, Barcelona) based on the glycerol phosphate oxidase/peroxidase method. Plasma samples (10 μl) were run in duplicate, and the concentration of triglyceride (milligrams per deciliter) was estimated from the sample absorbance at 500 nm. All samples were assessed in duplicate in two plates, with an average intra- and interplate CV of 4.9 and 10.6%, respectively.

### DNA extraction and molecular sexing

We extracted DNA from red blood cells from 1-day-old chicks by using a commercially available kit (Quick-DNA Miniprep Plus Kit; D4069; Zymo Research; USA) and following the manufacturer’s instructions. Gull chicks were sexed following the methodology and primer sequences described in (*68*). This methodology is based on polymerase chain reaction to amplify part of the W-linked avian *CHD* gene (*CHD-W*) in females and its non-W-linked homolog (*CHD-Z*) in both sexes. The DNA products were run on a 2% agarose gel and stained with Greensafe Premium (NZYtech, Portugal).

### Global DNA methylation

DNA methylation is the most widely studied epigenetic mark and involves the addition of a methyl group (-CH3) to the 5′ carbon of cytosines, primarily at CpG nucleotides. Changes in DNA methylation play a key role in promoting chromatin stability and regulating the expression of associated genes, although the direction of the effect depends on the genomic location. While methylation at CpG-rich regulatory regions has been typically associated with gene silencing, DNA methylation within the gene body has been shown to be positively correlated with gene expression (*69*). DNA methylation in peripheral tissues is often correlated with the epigenetic patterns found in other tissues, such as the brain or bone (*70*, *71*), but see (*72*). Peripheral blood is used to assess environmentally induced epigenetic changes in genes associated with individual responses to stress (*25*, *73*, *74*). DNA methylation patterns have been found to change in response to environmental cues in both captive (*24*, *73*) and wild (*23*, *25*) birds, which suggests that DNA methylation plays a crucial role in regulating developmental trajectories and phenotypic outcomes.

We estimated the presence of 5-methylcytosine (5-mC) in the genome by using a commercial ELISA kit (5-mC DNA ELISA kit; D5325; Zymo Research, USA). Briefly, 100 ng of DNA per sample was denatured at 98°C for 5 min and subsequently treated with an anti-5-mC monoclonal antibody. Afterward, DNA samples were treated with a secondary antibody containing horseradish peroxidase and a color developer. After 20 min of incubation at room temperature, the absorbance of each sample was measured at 450 nm in a Synergy 2 Multi-Mode Microplate Reader (BioTek Instruments Inc.). Levels of global DNA methylation were expressed as a percentage of 5-mC in each DNA sample (100 ng) estimated from a standard curve generated with specially designed DNA standards of known 5-mC percentage (0 to 100%). All samples were run in duplicate in two plates, with an average intra- and interplate CV were 10.7 and 13.6%, respectively.

### Statistical analyses

All analyses and graphs were performed using R version 4.1.2 (*75*). Assumptions of homoscedasticity and normality were verified by the inspection of diagnostic plots for residuals. Variables were transformed (natural logarithm [log] or square root, see below), when appropriate, to meet model requirements. We used linear models to assess initial between-groups differences in egg volume and laying date and generalized linear models (GLMs; binomial error distribution) to explore initial differences in sex ratio. Egg volume, laying date, and sex ratio did not differ between the experimental treatments (all *P* > 0.62). We also used a GLM (quasibinomial distribution to deal with overdispersed data) to assess the link between incubation constancy (proportion of time that the clutch remained unattended, i.e., more than 50% of maximum light intensity) and parent-parent communication. Confident intervals of repeatability estimates for parent-parent vocal communication during incubation were calculated via parametric bootstrapping (2000 iterations) using the rptR package (*76*).

The effects of our embryo treatment on developmental time, hatchling mass, global DNA methylation, and begging intensity (pecking behavior) at day 1 posthatching were analyzed through separate linear mixed models (LMMs) by using the lme4 package (*77*). Our models included the embryo treatment (embryo exposure to parent-parent vocal communication), embryo sex (female/male), hatching order (egg A/egg B), egg volume, and laying date (Julian day) as fixed effects. The two-way interaction between embryo treatment and sex, as well as the interaction between the genetic parents’ vocal communication and embryo treatment, was also included as a fixed term. The effects of embryo treatment on plasma CORT levels at day 1 posthatching were also analyzed through LMMs, including embryo treatment, sampling time (<3 min or 30 min), chick sex, hatching order, egg volume, laying date, and the two-way interactions between embryo treatment and sampling time as fixed effects. All LMMs above included both incubator ID and brood ID as random factors. Sample ID was also included as a random factor in our CORT model.

We also used LMMs (lme4 package) to test the effects of our embryo treatment on chick growth rate and plasma triglyceride levels at day 8 posthatching, as well as on offspring growth at the end of the growth period (tarsus length and square root transformed body mass at day 30 posthatching). These models included our embryo treatment, foster parents’ vocal communication (i.e., the total duration of parental vocalizations in seconds, log-transformed), genetic parents’ vocal communication (log-transformed), begging intensity, embryo sex, brood hierarchy, egg volume, and laying date as fixed effects. The two-way interaction between the genetic parents’ vocal communication and embryo treatment or foster parents’ communication was included as a fixed factor. The three-way and two-way interactions between embryo treatment, foster parents’ vocal communication, and sex were also included as fixed factors. Incubator ID and brood ID were included as a random factor in all models. For all models described above, we report standardized coefficients (95% CI) from full models after removing nonsignificant interaction terms (*78*). The quality of fit of all models was evaluated by using the performance package (*79*). Models with singular fits (CORT levels, begging intensity, growth rate, triglyceride levels, and tarsus length models) were refitted using the glmmTMB package (*80*) with specified Gamma priors (*81*). *P* values were obtained from Wald statistics by using the car package (*82*). Marginal effects from regression models were plotted by using the visreg package (*83*). All values are presented as mean ± SE.
