# Supplementary material for: Lasting benefits of embryonic eavesdropping on parent-parent communication
Source: Sci Adv. 2024 Aug 30;10(35):eadn8542. doi: 10.1126/sciadv.adn8542 (PMC11364100; doi:10.1126/sciadv.adn8542)
Supplement: Supplementary file 1 — Figs. S1 to S7 Tables S1 to S3 Legends for movies S1 to S3 [file sciadv.adn8542_sm.pdf]

Science Advances
AAAS

Supplementary Materials for

Lasting benefits of embryonic eavesdropping on parent-parent communication

Francisco Ruiz-Raya and Alberto Velando

Corresponding author: Francisco Ruiz-Raya, francisco.ruiz-raya@glasgow.ac.uk

Sci. Adv. **10**, eadn8542 (2024)
DOI: 10.1126/sciadv.adn8542

The PDF file includes:

Figs. S1 to S7
Tables S1 to S3
Legends for movies S1 to S3

Other Supplementary Material for this manuscript includes the following:

Movies S1 to S3

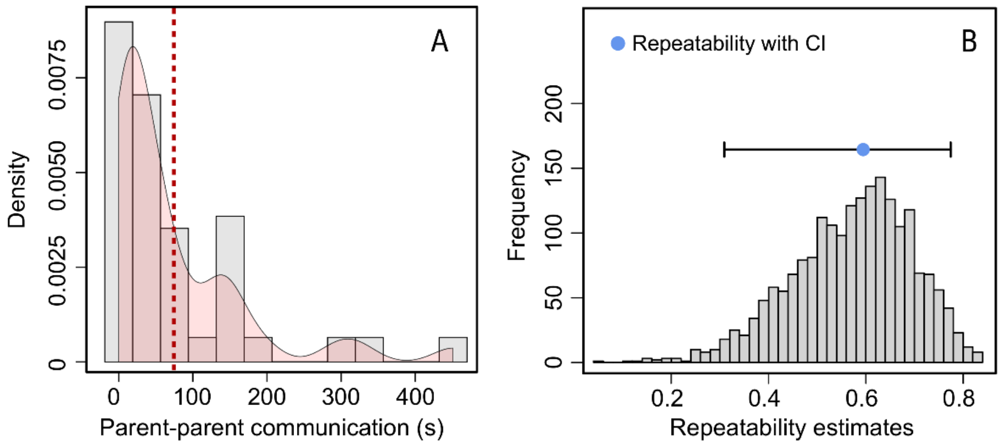

Fig S1. A. Distribution of gull pairs according to their levels of parent-parent vocal communication (vocal communication/day; in seconds) during late incubation (embryonic day 24; N = 44). The dotted red line denotes the mean value of the population (71.8 ± 10.6 s). **B.** Within-pair bootstrap repeatability (with confidence intervals, CI) for parent-parent vocal communication (embryonic day 24 vs 26; N = 35). Levels of parent-parent vocal communication were highly repeatable within experimental families throughout late incubation (R = 0.594 ± 0.12 [0.314, 0.777], *P* = 0.0002).

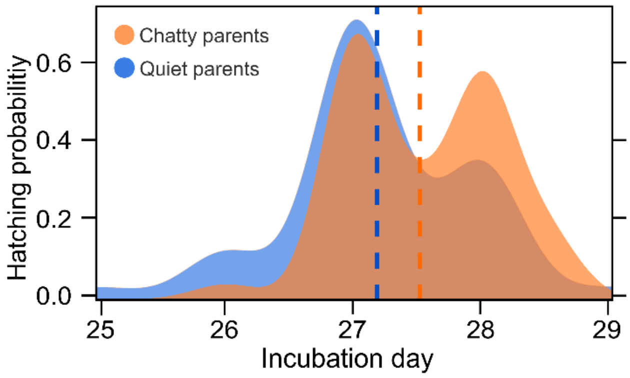

Fig. S2. Hatching probabilities for gull embryos exposed to different levels of parent-parent vocal communication. Developmental period was shorter on average in embryos exposed to acoustic cues of quiet parents compared to embryos exposed to prenatal acoustic cues of chatty parents (LMM: β=-0.53, 95% CI: -0.96 to -0.11, *P* = 0.012).

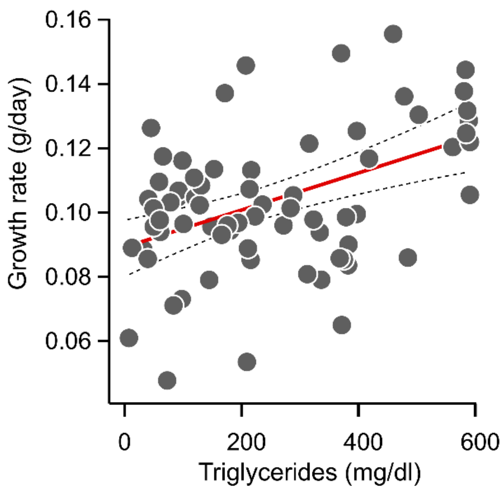

Fig. S3. Correlation between growth rate and lipid profiles (plasma triglyceride levels) in 8 days-old gull chicks (R = 0.42; *P* < 0.001).

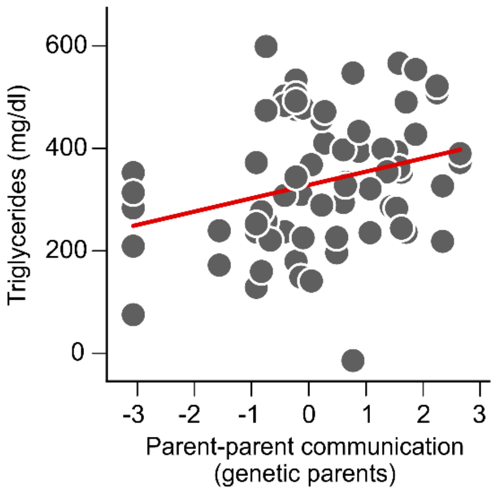

Fig. S4. Positive relationship between plasma triglyceride levels in 8-day old chicks and intra-pair communication showed by genetic parents (LMM: *begging intensity:* β=0.24; 95% CI: 0.03, 0.44; *P* = 0.021). Mean-centered values for (genetic) parent-parent communication are shown.

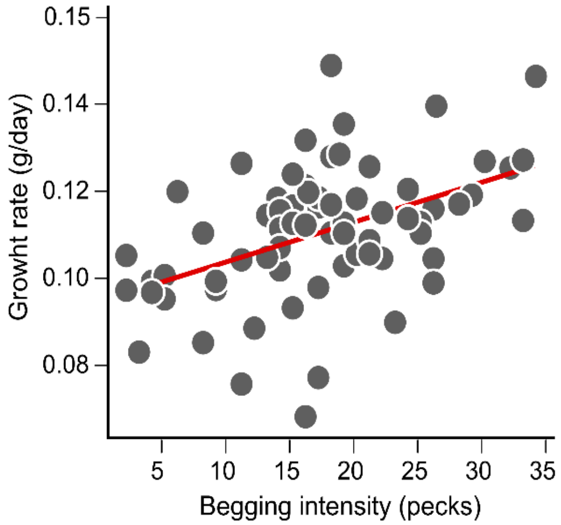

Fig. S5. Relationship between offspring growth and food solicitation during early postnatal life: growth rate during the first 8 days after hatching was positively related to begging (pecking behaviour; LMM: *begging intensity:* β=0.32; 95% CI: 0.11, 0.54; *P* = 0.002).

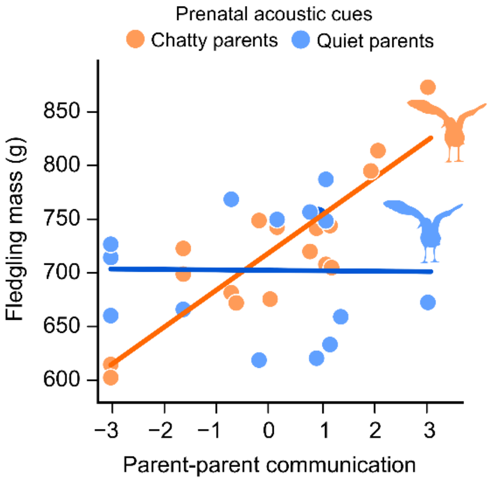

Fig. S6. Lasting benefits of embryonic eavesdropping. Body mass at day 30 post-hatching was positively related to (foster) parental communication levels, but only for those chicks that were prenatally exposed to acoustic cues of chatty parents (LMM: *embryo treatment x foster parent communication:* β = -0.56; 95 % CI: -1.12, -0.02; *P* = 0.034). Mean-centre values for parent-parent communication are shown.

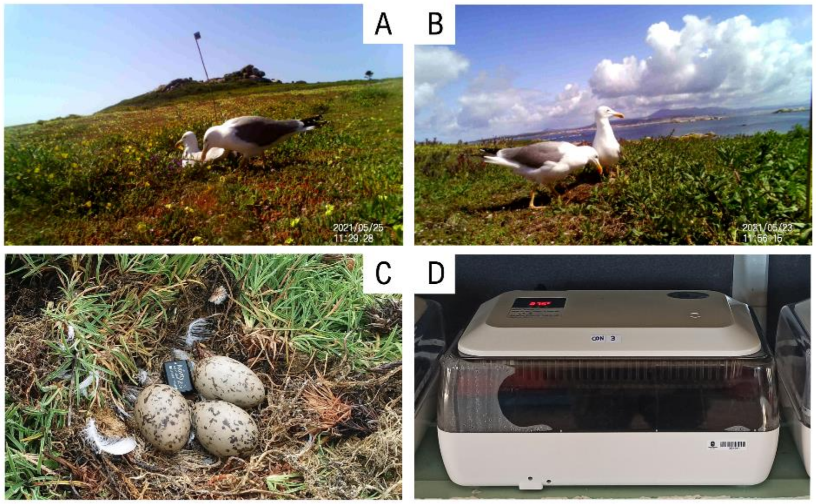

Fig. S7. **A.** Encounter between experimental parents during incubation: the incoming individual approaches the nest while its partner is incubating (note that the incoming individual adopts the typical choking posture while vocalising). **B.** Nest relief: the incubating adult leaves the nest and the incoming individual initiates incubation. **C.** Dattaloger device used to assess natural variations in light intensity between yellow-legged gull clutches as a proxy of incubation efficiency. **D.** Detail of one of the six artificial incubators used during the embryo treatment (from incubation day 22 to 28). Photo Credits: Francisco Ruiz-Raya, University of Glasgow.

Table S1.

Summary of LMMs testing the effects of prenatal acoustic cues of parental coordination on embryo developmental time, hatchling mass, global DNA-methylation, corticosterone levels, and begging intensity (pecking behavior) at day 1 post-hatching. Significant terms are highlighted in bold.

a)	Developmental time		Hatchling mass	
Source of variation	*β (95% CI)*	*P-value*	*β (95% CI)*	*P-value*
Embryo treatment (*quiet parents*)	-0.43 (-0.80, -0.08)	**0.015**	-0.06 (-0.30, 0.18)	0.620
Sex (*male*)	-0.39 (-0.74, -0.04)	**0.028**	-0.12 (-0.39, 0.15)	0.391
Hatching order (*B-egg*)	0.45 (0.16, 0.75)	**0.002**	-0.14 (-0.37, 0.10)	0.255
Egg volume	0.01 (-0.22, 0.22)	0.995	0.77 (0.61, 0.92)	**<0.0001**
Hatching date	-0.06 (-0.33, 0.21)	0.661	-0.08 (-0.25, 0.08)	0.282
Random effects	*Variance*		*Variance*	
Incubator ID	0.01		0.02	
Brood ID	0.22		2.59	
Residual	0.20		5.50	
	$R^2m = 0.13$		$R^2m = 0.59$	
	$R^2c = 0.60$		$R^2c = 0.72$	

b)	Global DNA-methylation	
Source of variation	*β (95% CI)*	*P-value*
Embryo treatment (*quiet parents*)	0.47 (0.03, 0.90)	**0.032**
Sex (*male*)	-0.12 (-0.58, 0.34)	0.592
Hatching order (*B-egg*)	-0.17 (-0.59, 0.25)	0.426
Egg volume	0.17 (-0.07, 0.41)	0.148
Hatching date	0.03 (-0.21, 0.27)	0.810
Random effects	*Variance*	
Incubator ID	0.05	
Brood ID	0.84	
Residual	6.42	
	$R^2m = 0.08$	
	$R^2c = 0.19$	

c)	Corticosterone levels	
Source of variation	*β (95%CI)*	*P-value*
Embryo treatment (*quiet parents*)	-0.06 (-0.47, 0.35)	0.332
Sampling time (30 min)	0.87 (0.56, 1.18)	**<0.0001**
Sex (*male*)	-0.22 (-0.49, 0.06)	0.123
Hatching order (*B-egg*)	-0.12 (-0.36, 0.12)	0.331
Egg volume	-0.01 (-0.16, 0.14)	0.864
Hatching date	-0.13 (-0.33, 0.07)	0.213
Embryo treatment x Sampling time	0.46 (0.02, 0.89)	**0.039**
Random effects	*Variance*	
Incubator ID	316.5	
Brood ID	851.5	
Sample ID	357.3	
Residual	4023.2	
	$R^2m = 0.19$	
	$R^2c = 0.40$	

d)	Begging intensity	
Source of variation	*β (95% CI)*	*P-value*
Embryo treatment (*quiet parents*)	-0.54 (-1.03, -0.04)	**0.022**
Sex (*male*)	-0.01 (-0.45 0.42)	0.974
Hatching order (*B-egg*)	0.13 (-0.28, 0.53)	0.575
Egg volume	-0.09 (-0.32, 0.13)	0.509
Hatching date	-0.01 (-0.26, 0.24)	0.986
Random effects	*Variance*	
Incubator ID	0.31	
Brood ID	0.35	
Residual	54.10	
	$R^2m = 0.19$	
	$R^2c = 0.40$	

Table S2.

Summary of LMMs testing the effects of prenatal acoustic treatment of parental coordination on offspring growth rate and nutritional condition (plasma triglyceride levels**)** during the first 8 days of life. Significant terms are highlighted in bold.

	Growth rate		Triglyceride levels	
Source of variation	*β (95% CI)*	*P-value*	*β (95% CI)*	*P-value*
Embryo treatment (*quiet parents*)	-0.63 (-1.04, -0.22)	**0.009**	-0.81 (-1.22, -0.40)	**<0.0001**
Foster parent communication	0.34 (0.06, 0.63)	0.269	0.52 (0.24, 0.80)	**0.003**
Genetic parent communication	0.06 (-0.16, 0.28)	0.598	0.24 (0.03, 0.44)	**0.012**
Begging intensity	0.33 (0.13, 0.52)	**0.001**	0.05 (-0.15, 0.25)	0.680
Sex (*male*)	0.01 (-0.38, 0.40)	0.908	0.01 (-0.39, 0.40)	0.944
Hatching order (*B-egg*)	0.09 (-0.25, 0.42)	0.604	-0.19 (-0.55, 0.16)	0.315
Egg volume	-0.06 (-0.27, 0.16)	0.642	-0.17 (-0.37, 0.04)	0.073
Hatching date	-0.14 (-0.38, 0.09)	0.250	-0.03 (-0.24, 0.19)	0.702
Embryo treatment x Foster communication	-0.41 (-0.75, -0.06)	**0.020**	-0.45 (-0.81, -0.09)	**0.019**
Random effects	*Variance*		*Variance*	
Incubator ID	9.95e-05		0.36	
Brood ID	1.42e-05		0.35	
Residual	2.21e-05		1.18	
	$R^2m = 0.39$		$R^2m = 0.37$	
	$R^2c = 0.39$		$R^2c = 0.37$	

Table S3.
Summary of LMMs testing the effects of prenatal acoustic cues of parental coordination on body size (tarsus length) and mass at day 30 post-hatching. Significant terms are highlighted in bold.

	Tarsus length		Body mass	
Source of variation	*β (95% CI)*	*P-value*	*β (95% CI)*	*P-value*
Embryo treatment (*quiet parents*)	-0.48 (-0.87, -0.09)	**0.043**	-0.07 (-0.31, 0.18)	0.786
Foster parent communication	0.46 (-0.05, 0.86)	0.269	0.20 (0.01, 0.38)	0.129
Genetic parent communication	-0.25 (-0.62, 0.13)	0.145	-0.07 (-0.21, 0.07)	0.298
Begging intensity	-0.20 (-0.39, 0.01)	0.145	0.04 (-0.08, 0.17)	0.768
Sex (*male*)	0.07 (-0.35, 0.50)	0.760	0.09 (-0.16, 0.35)	0.515
Hatching order (*B-egg*)	-0.12 (-0.37, 0.14)	0.433	-0.06 (-0.26, 0.14)	0.576
Egg volume	0.28 (-0.09, 0.64)	**0.031**	0.17, (0.02, 0.32)	**0.030**
Hatching date	-0.04 (-0.38, 0.30)	0.929	0.01 (-0.14, 0.16)	0.889
Embryo treatment x Foster communication	-0.49 (-0.77, -0.20)	**0.002**	-0.25 (-0.47, -0.04)	**0.009**
Random effects	*Variance*		*Variance*	
Incubator ID	0.18		0.10	
Brood ID	6.58		0.96	
Residual	1.81		1.14	
	$R^2m = 0.61$		$R^2m = 0.32$	
	$R^2c = 0.62$		$R^2c = 0.64$	

Movie S1.

Example of nest relief during incubation in which parents show low levels of vocal communication.

Movie S2.

Example of nest relief during incubation in which parents show high levels of vocal communication.

Movie S3.

Food distribution among yellow-legged gull (*Larus michahellis*) chicks. In this species, provisioning rules are mainly controlled by the parents, who may show preferences when distributing food.
